# Erratum to “Tumour volume distribution can yield information on tumour growth and tumour control” [Z Med Phys 32 (2022) 143–148]

**DOI:** 10.1016/j.zemedi.2024.07.010

**Published:** 2024-08-28

**Authors:** Uwe Schneider, Jürgen Besserer

**Affiliations:** a Department of Physics, Science Faculty, University of Zürich, Zürich, Switzerland; b Radiotherapy Hirslanden, Witellikerstrasse 40, CH-8032 Zürich, Switzerland

The publisher regrets that the declaration of competing interest statement was not included in the original article. The correct declaration of competing interest statement for this article is:

The authors declare that they have no known competing financial interests or personal relationships that could have appeared to influence the work reported in this paper.

The publisher would like to apologise for any inconvenience caused.

